# Investigation of Nursing Students’ Verbal Communication Quality during Patients’ Education in Zahedan Hospitals: Southeast of Iran

**DOI:** 10.5539/gjhs.v8n9p331

**Published:** 2016-02-01

**Authors:** Fatemeh Kiani, Abbas Balouchi, Alireza Shahsavani

**Affiliations:** 1 Health Promotion Research Center, Zahedan University of Medical Sciences, Zahedan, IR Iran; 2 Student Research Committee (SRC), School of Nursing and Midwifery, Zabol University of Medical Sciences, Zabol, IR Iran

**Keywords:** students, nursing, patients, education, verbal, communication

## Abstract

**Background:**

Most basic nursing skill is communicating with the patient. Nurses must be able to provide their professional services well for patients while communicating with them therefore examining the professional skills of nursing students as those who make the future nursing community is of great importance.

**Objective:**

The aim of this study was Investigation of nursing students’ verbal communication quality during patients’ Education in Zahedan hospitals: southeast of Iran

**Materials and Methods:**

A cross-sectional study was conducted on 95 nursing students in two Educational hospitals of Zahedan, Iran from November 2013 through March 2014.sampling method was census. Researcher made checklist was used to gather the data. Statistical tests of frequency distribution, mean, SD and chi-squire were used to analyze the data.

**Results:**

Most of the students in the start N=45 (47.4%) and during N=48 (50.5%) of verbal communication with the patients had the good verbal communication but in the end of communication the patients most students N=33 (34.7%) had average verbal communication and N=31 (32.6%) of them had poor verbal communication.

**Conclusion:**

Since quality of verbal communication in the end of patient education is poor and good communication between the patient and nurse is the basic component of patient care and its Educational plans should be coordinated with clinical practices and be parallel to it, also effective methods must be used.

## 1. Introduction

Communication is one of the basic needs of humans. All the professions need communication skills but no one needs such a complicated communication as that of nursing because nurse is someone who should play different roles daily due to contact with patients (Hemsley et al., 2012). Apply communication principles plays a important role in nursing because at first nurses’ task in the treatment is comply primary needs of patients by correct communicating (Carol et al., 2003). It is necessary for nurses to be able to have proper communication with patients, patients’ family and health care workers to provide Suitable their professional care (McCabe, 2004) Nursing students are of those groups who learn communication skills by faculties, these skills Education affects their performance significantly and its effect can be observed in patients’ satisfaction rate (Lin et al., 2013; Sabzevari et al., 2006). The aim of practical Education plans is increasing basic communication skills. A study showed that the students did not have enough communication skills and they only communicate with patients while providing services and cares thus a more effective Education for students have been emphasized (Zamanzadeh et al., 2014). Effective communication causes health promotion of patients and also satisfaction increase. Communication failure leads to wrong diagnosis, decreases patients’ contribution in treatment and lessens providing information for the patients (Greco, 2002). Educational plans should be parallel and coordinated to clinical practices also effective Educational methods should be utilized (Schlegel et al., 2012). Nurses need to communicate with patients to apply their educational role (Tay et al., 2011). Nurses communicate with patients in a short time; they speak with them superficially about physical problems and do not attend care aspects (Greco, 2002). Factors like lack of enough training of principles and skills of communication to nurses during education, not holding educational course about communication and its skills for nurses, having more than one job and its fatigue can impede effective communication (Garrett et al., 1996). Only few studies related to nurses student communication skills are done in Iran and these skills training studies aimed to satisfy the patients are also few (Yaghoubi, & Rahmati-Najarkolaei, 2014).

## 2. Objective

This study aimed to Investigation of nursing students’ verbal communication quality with patients during patients’ Education in Zahedan hospitals: southeast of Iran in 2014.

## 3. Materials and Methods

### 3.1 Design

A cross-sectional study was conducted on 95 nursing students who were studying the third semester or more in two Educational hospitals of Zahedan University of medical sciences (from six different possible hospital) from November 2013 through March 2014. Zahedan is the center of Sistan v Baluchistan State that it is largest State of Iran that hold in southeast of Iran. Sampling method was Census. Inclusion criteria were satisfaction for participate and verbal communication ability.

### 3.2 Instrument

Data was collected by used a researcher made check list. This check list was finalized after repeated discussion with experts, using other studies that were similar to this study (McCabe, 2004; Hemsley et al., 2012; Bambini et al., 2009) and a pilot study. The check list include of 3 parts: The first part-demographic information-Age (year), Gender (male/female), marriage (single/married), residential status (Dormitory/No dormitory), semester (4-6-7) and interest to nursing (Yes/No). The second part provided a checklist for assessment of communications skills in three sub domain start, during and end of communicates with patient. First sub domain was start of communication with the patient that included 7 multiple choice questions. Every question has 3 options that scoring were defined poor (1), average (2) and good (3). The total score of start of communication was between 7 to 21 scores. Scores less than 10 (poor), 10 to 15 (average) and 16 or higher were considered good. The second sub domain was assessed communication skills during of communication with the patient that included 11 multiple choice questions. Scoring of every question was as start of communication. The total score of during of communication was between 11 to 33 scores. Scores less than 15 (poor), 16 to 25 (average) and 26 or higher were considered good. The third sub domain was assessed communication skills at the end of communication with the patient that included 4 multiple choice questions. Scoring of every question was as previous parts. The total score of end the communication was between 4to 12. Scores less than 5 (poor), 6 to 9 (average) and 10 or higher were considered good. Content validity of questionnaire was approved by 10 nurse instructors, and 3 psychologists. Reliability questionnaire was approved Cronbach’s alpha method after administering on 20 nursing students. Cronbach’s alpha was 0.84. A complete census was planned to registration the appropriate sample size. First aim and data gathering were explained to the instructors. For data gathering and increase of data validity students behaviors were observed in the start, during and at the end of patients’ Education. A specific instructor was supposed to observe only one student. Because gender affects communication according to the results of other studies and also obeying religious laws, patients of the same gender was selected for every student.

### 3.3 Ethical Consideration

This study was approved by ethics committee of Zahedan University of Medical Sciences. Written informed consent was obtained from all students.

### 3.4 Statistical Analysis

SPSS (SPSS Inc. in Chicago V16) statistical software was employed for data analysis to describe data; the statistical tests of frequency distribution, frequency percentage, mean, and standard deviation and chi-squire were used. Significant statistical significance was considered at p<0.05.

## 4. Results

All students contributed and replication rate was 100%. This study finding showed mean and Standard deviation of participants’ age were 23±0.9. Most of participants were females N=51 (53.7%), single N=78 (82.1%), No residence of dormitory N=48 (50.5%), studying 4^th^ semester N=35 (36.8%) and interested to nursing N=63 (66.5%) (Table 1).

**Table 1:** Demographic characteristics of nursing students

Variables		N	%
**Gender**	Male	44	46.3
	Female	51	53.7
**Marital status**	Single	78	82.1
	Married	17	17.9
**Residence**	Dormitory	47	49.5
	Non dormitory	48	50.5
**Semester**	4	35	36.8
	6	29	30.5
	7	31	32.7
**Interest to nursing**	Yes	63	66.3
	No	32	33.7

**Figure 1:**
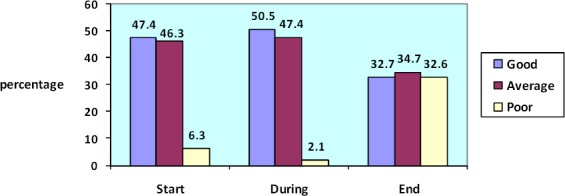
Quality of verbal communication of nursing students in start, during and end of communication

Most of the students in the start of verbal communication with the patients N=45 (47.4%) had the good verbal communication and only N=6 (6.3%) of them had poor verbal communication. Most students N=48 (50.5%) of them had the good verbal communication during communication with patients and only 2 students (2.1%) had poor verbal communication but at the end of communication the patients most students N=33 (34.7%) had average verbal communication and N=31 (32.6%) of them had poor verbal communication. Most students of this study had better communication in during of patient education. (Figure 1)

There was no significant relationship among gender, marriage, residential conditions and interest rate with quality of verbal communication during patient education (p<0.05). Results of Chi-squire test showed that there is a significant relationship between verbal communication quality and semester in the start and during of patients’ Education, but there was no Relationship at the end of Education between semester and verbal communication quality (Table 2).

**Table 2:** Relationship between quality of communication and semester at start, duration and end of the communication with patients

Steps	Semester	Quality of communication						
		Good		Average		Poor		P value
**Start**	4	11	31.4	21	60	3	8.6	P=0.016
	6	21	72.4	8	27.6	0	0	
	7	13	41.9	15	48.4	3	9.7	
**During**	4	10	28.6	23	65.7	2	5.7	P=0.03
	6	21	72.4	8	27.6	0	0	
	7	17	54.8	14	45.2	0	0	
**End**	4	9	42.9	11	31.4	15	25.7	P=0.24
	6	7	24.2	9	31	13	44.8	
	7	31	31.7	33	34.7	31	32.6	

## 5. Discussion

Our finding shows that less than half of the nurse’s students 47.4% have good communication with patients in start of communication that MA Hajbaghery et all study confirmed it (Hajbaghery, & Shahsavarloo, 2014), but results of the study of Taheri et al. showed that most nursing students accept patient education and consider it as important duty of nurses (Tahery et al., 2011). Studies also indicate that instructing the patients is of great importance from the nurses’ score of view. Patient education has many effects like client satisfaction increase, quality of life improvement, decrease of diseases complications, treatment costs and hospital reception. Half of them had the good verbal communication during instructing the patients and 32.6% of them had the good verbal communication at the end. Results (Brock et al., 2013; Henderson et al., 2012) showed that verbal communication quality at the end is lower than the start and during of Education. We can see items at the end of interview which are used less in daily conversation which may be the reason of lower scores at the end of discussion. Most of the studies have discussed effective factors on instructing the patients or its benefits (Ito et al., 2014; Schulz & Nakamoto, 2013). Certainly quality of verbal communication is one of the important factors of instructing the patients. Since public attitudes about providing essential information and educational needs has been changed in recent decades so medical and health team members are expected to present enough information about disease, its complications, treatment and self-care for patients and their families finally patients become aware of disease aspects and treatment procedures and can have more active and independent role in their own decision making (Coster, & Norman, 2009). It can be achieved by interaction and effective relationships. Most of the students (51.8%) agreed the nurses’ effective relationship role with patients regarding patients’ acceptance (Tahery et al., 2011). The results of study showed that there was no difference between male and female students regarding verbal communication quality however it seems that women are more successful in communication than the mans. Studies mention that women’s verbal communication capability or their skill in using language is more than men. Women’s tone is more variable than that of men and they speak with a more variable tone. There is no obvious difference between spoken words of men and women but generally women speak better and more grammatical in the same cultural and educational conditions. Conversation and opinion exchange is by information exchange for most men while this is through sympathy and emotional affairs for women (Brody, & Hall, 2008). Also results showed that marital status also has no effect on communication while other studies show that married have better and more communicational skills than singles which indicates the effect of psychological features in communicational skills (Behrad, 2011).

Results showed that there is a significant relationship between semester and verbal communication quality so 6th semester students had a better verbal communication quality which was significant in the start and during the interview but it was decreased at the end. Studies indicate that students’ communicational skills increase as their education progresses to the end (de Azevedo et al., 2015). This study totally showed that those nursing students who were interested in nursing had a better verbal communication quality than those who were not interested in nursing, but statistical tests did not show the significance. Certainly motivation is the first factor for Education and proper communication regarding determining success in Education (Stuart, 2014). Aieen’s et al study showed that students have more opportunity to contact the patients which leads to more precise diagnosis of their problems and response but lack of enough communicational skills results in patients’ distrust, dissatisfaction and sometimes complaining from ward nurses (Aein et al., 2010). Results of the study of Azizi Nejad showed that nurses’ lack of appropriate communicational skills with clients was one of the setbacks of patient education according to 70% of nurses Viewpoint (Aziznejad et al., 2010). Celik has stated that unawareness of nurses and inappropriate communication with patients are of the most important obstacles of patient education (Celik et al., 2008). Results of the study of Taheri showed that facilitative Education factors, communicational skills, informative awareness and interest are so effective (Tahery et al., 2011). Other studies also prove this theory (Aziznejad et al., 2010). It should be attended that it is possible by interaction and effective communication (Nordgren, & Fridlund, 2001). Communicational skills are not embedded into the official medical syllabus of Iran obviously so effective communication, responding to questions and solving patients’ needs by physicians and nurses has encountered weaknesses and defects in many cases (Zamani et al., 2004), which can due to from Negligence the essential and vital role of communication in therapeutic process between patients and medical team members (Arbabisarjou et al., 2015). Patients feel that they have communicated effectively with medical team which enables them to state their tendencies and Educational needs and to prepare the condition to be ready to listen and learn Educational materials (Rad & Ashari, 2004). It can be recommended based on above mentioned scores to have more emphasis on in patient education in nursing courses and course topics should be taught in details in nursing colleges. Nursing trainers with students should design and execute practical work in internship and novitiate courses. It is also recommended that both hospitals and nursing colleges deans try to help nurses obtain communicational skills and necessary knowledge of instructing the patients as reliable persons who can design, execute and evaluate Educational programs for patients (Sideras et al., 2013). This study has limitations, which must be addressed. This was a cross sectional study that reduced the ability of the study to make direct casual inferences. This study sample size was few due to the low number of nursing students in zahedan nursing faculty.

## 6. Conclusion

Although students had a good verbal communication quality in start and during patient education but it was not enough in end of communication. Recommended that another study about the effect of Education on the verbal communication quality of students should be carried out. Good communication between the patient and nurse is the basic component of patient care and its Educational plans should be coordinated with clinical practices and be parallel to it, also effective methods must be used.
